# Comparing Oral Versus Intravenous Antibiotics Administration for Cellulitis Infection: Protocol for a Systematic Review and Meta-Analysis

**DOI:** 10.2196/48342

**Published:** 2023-11-03

**Authors:** Raymond Yin, Jingyi Jiang, Yiyang Wang, Yuhao Jin, Eric Qian, Chenyang Yue, Coco Jiang, Michelle Wang, Kylie Xu, Xiaoyuan Zhou, Winston Hou

**Affiliations:** 1 Faculty of Science University of Western Ontario London, ON Canada; 2 Faculty of Science University of Toronto Toronto, ON Canada; 3 College of Life Sciences University of California, Los Angeles Los Angeles, CA United States; 4 Emory University Atlanta, GA United States; 5 Faculty of Science York University Toronto, ON Canada; 6 Faculty of Science Dawson College Montreal, QC Canada; 7 Faculty of Health Sciences McMaster University Hamilton, ON Canada

**Keywords:** antibiotics, cellulitis, infection, intravenous, oral

## Abstract

**Background:**

Cellulitis is defined as an infection of the skin that is usually characterized by localized but poorly demarcated areas of erythema, swelling, and pain. Erysipelas is a subtype of cellulitis that is characterized by a more superficial infection, often involving the face. Because gram-positive bacteria are the most common infective agent, beta-lactam antibiotics such as cephalosporins are commonly used. However, guidelines and physician preference vary widely as different antibiotic options and routes of administration exist, in addition to the fact that most cases are treated empirically without microbiological lab guidance. This lack of standardization in evidence, guidelines, and physician practice prompted this systematic review and meta-analysis of both randomized trial data and cohort studies to aggregate the currently available evidence for the optimal routes of antibiotic administration in cellulitis treatment.

**Objective:**

The primary objective of our review is to compare the efficacy of oral versus intravenous antibiotic administration for cellulitis infections, thereby providing clinicians with evidence-based guidelines for treatment.

**Methods:**

We will search MEDLINE, Embase, and CENTRAL through Ovid as well as Web of Science and CINAHL for all available literature comparing different routes of antibiotic administration in the treatment of cellulitis and erysipelas. We will perform title and abstract as well as full-text screening in duplicate according to the PRISMA (Preferred Reporting Items for Systematic Review and Meta-Analyses) guidelines and then extract the relevant data using a prepiloted data sheet. The primary outcome for our review is the duration of infection resolution, and secondary outcomes such as incidence of sepsis, mortality, hospital admission, and Clostridium difficile infection. We will assess the risk of bias in our included studies using the RoB 2.0 (revised tool for Risk of Bias in randomized trials) and ROBINS-I (Risk of bias in non-randomized studies for interventions) tools, with a final quality assessment using the GRADE (Grading of Recommendations, Assessment, Development, and Evaluation) framework and a sensitivity analysis to examine heterogeneity.

**Results:**

We will publish the final results of our systematic review in a peer-reviewed academic journal. This project received no funding or financial assistance. Data analysis is currently underway, and the results are expected to be submitted for publication in late November 2023.

**Conclusions:**

To our knowledge, this will be the most up-to-date review of the best available evidence comparing different routes of antibiotic administration for cellulitis. Because of the vast selection of antibiotic options available and the empirical nature of the treatment, we anticipate heterogeneity within our data but nonetheless hope to provide aggregated evidence on the efficacy of intravenous versus oral administration of antibiotics in cellulitis treatment. We hope the results from this study will better inform physician practices in the future for cellulitis infections.

**International Registered Report Identifier (IRRID):**

PRR1-10.2196/48342

## Introduction

Cellulitis is defined as a bacterial infection of the skin, usually involving the epidermal and dermal layers, with occasional involvement of the subcutaneous fat and lymphatic tissues. It is commonly characterized as poorly demarcated but localized swelling and pain, most often in the lower limbs [1]. Erysipelas is a subtype of cellulitis where the infection is more superficial in the epidermal layer and is often referred to by its clinical name when it affects the face or legs. The presentation of cellulitis also varies in severity and in bacterial species. Most patients only have mild systemic symptoms such as fevers, with only around 10% of hospitalized patients experiencing severe complications such as sepsis and necrotizing fasciitis [1,2]. However, around 18% of initial antibiotic treatment still fails. The most common causative agents include beta-hemolytic streptococci, such as group A and group G, as well as *Staphylococcus aureus*. The presence of methicillin-sensitive versus methicillin-resistant species of *S aureus* is also crucial in directing antibiotic treatment [2]. However, because of the nonsterile site of cellulitis infection with the presence of numerous commensal bacteria, empiric treatments are usually started without specimen collection. Microbiological studies are often only conducted in a research setting, to a limited effect, as the presence of commensal colonies can often confound the culture results. In rare cases, through opportunistic infections, fungal species can be implicated as well [3].

Based on the prominent bacterial species possibly found in common cellulitis infections such as beta-hemolytic streptococci and staphylococci, gram-positive antibiotics covering these organisms have been the empiric drug of choice [3,4]. However, a wide range and different classes of antibiotics are available to fill this demand, and there have been no cohesive guidelines for standardization. Beta-lactam inhibitors such as penicillin or different generations of cephalosporins such as cefazolin, cephalexin, or ceftriaxone have been commonly used. Other classes of antibiotics such as macrolides, doxycycline, and clindamycin have all been used and reported in the literature [3]. Additionally, antibiotics used for the broader coverage of methicillin-resistant *S aureus*–related cellulitis also vary, from vancomycin to trimethoprim-sulfamethoxazole or clindamycin. These antibiotics all differ in their efficacy and side effect profiles, such as *Clostridium difficile* risks. This lack of standardization and reliance on physician preference points to the need for evidence-based reviews for the optimal treatment of cellulitis [3-5]. These antibiotic options also differ in their routes of administration, with some available in both oral and intravenous (IV) routes, while other antibiotics can only be administered through one of the options. Because of its potential involvement in deeper layers of skin, topical antibiotic administration is unlikely to show efficacy against the infection. Despite some evidence showing that oral therapies are at least as effective as IV administration, some guidelines still recommend the IV routes for high-risk patients, such as those with obesity or venous stasis [3,6].

It is for these reasons mentioned above that we have decided to perform this systematic review. The primary objective of our review is to compare the efficacy of oral versus IV antibiotic administration for cellulitis infections, thereby providing clinicians with evidence-based guidelines for treatment.

## Methods

### Overview

This systematic review and meta-analysis will be conducted in accordance with the PRISMA (Preferred Reporting Items for Systematic Review and Meta-Analyses) guidelines [7]. This protocol is also registered with PROSPERO (International Prospective Register of Systematic Reviews; registration ID CRD42023413590). Any major changes to this protocol will be reported within the final review itself.

### Search Methods

We will conduct a systematic search of MEDLINE, Embase, and CENTRAL through Ovid, as well as CINAHL and Web of Science, from inception to February 2023. We will also use medical subject headings terms, when possible, for broad inclusion of studies. Examples include “erysipelas.tw,kf.” and “(antimicrob* or antibiotic*).tw,kf.” A detailed sample search strategy for MEDLINE through Ovid is outlined in Multimedia Appendix 1. We will not have any language restrictions on the data in our search. The bibliography of previous literature and systematic reviews will also be hand-searched for relevant or potentially missed articles.

### Eligibility Criteria

#### Types of Studies

We will include parallel group randomized controlled trials (RCTs) as well as both prospective and retrospective cohort studies. Studies with skin and soft tissue infection as its main focus and cellulitis as one of its subgroups will also be included in the review. Case control, case series, and case reports will be excluded. Studies focusing on other skin conditions such as necrotizing fasciitis with noncellulitis sources will also be excluded. The detailed inclusion and exclusion criteria are organized into a table format in Table S1 in Multimedia Appendix 2.

#### Types of Participants

Our review will focus on all adult patients (aged 18 years or older) who were diagnosed with cellulitis or erysipelas in any health care setting. Cellulitis diagnostic criteria are based on individual studies.

#### Type of Intervention

We will include all studies comparing oral versus IV administration of antibiotics for the treatment of cellulitis. The antibiotic agent does not have to be the same across the different treatment groups within the study, nor does it have to be from the same class. For example, if a study compares IV cephalosporin versus an oral macrolide, it will be included in our analyses. Studies comparing antibiotics against placebos or other types of alternative treatments will be excluded. We are only focusing on antibiotics; hence, other rarer causes of cellulitis such as fungal infections will be excluded.

### Outcomes

The primary outcome of our review will focus on the duration of infection resolution, which may vary across studies and may need to be adjusted based on our included articles’ definition of infection resolution. Our second outcomes include incidence of severe systemic infection or mortality, patients’ quality of life (QOL) as measured by QOL scores based on each included study, incidence of adverse events such as nausea and vomiting, length of hospitalization for inpatients or number of hospitalizations for cellulitis in studies measuring outpatients, and incidence of *C difficile* infection.

### Study Screening

A total of 2 independent authors will perform title and abstract screening individually and in duplicate using Covidence, a web-based systematic review tool [8]. If both reviewers are in agreement, a study will move on to full-text screening. Any conflicts between reviewers will be resolved through discussion involving a third author. Full-text screening will also be conducted in the same manner. If a full text cannot be found on publicly available domains, attempts will be made to reach out to the authors for a potential copy of the article with relevant data.

### Data Collection

All data will be collected using a priori developed data collection sheet within Excel (Microsoft Corp), with 2 reviewers working independently and in duplicate, resolving conflicts through discussion and third-party arbitration. The prepiloted forms will be tested using 10 of the first included studies. For any full texts that are not available in the public domain or databases and for any missing data, we will attempt to contact the authors for further clarification and assistance.

### Risk of Bias Assessment

The risk of bias (RoB) assessments of each analyzed study will be performed by 2 independent reviewers in duplicate. The RoB of RCTs will be assessed through RoB 2.0, a revised tool to assess RoB in randomized trials [9]. The reviewers will assess bias across the 5 outlined domains: bias arising from the randomization process, from deviation from intended interventions, from missing outcome data, from the measurement of outcomes, and from the selection of reported results. The overall RoB will be compiled using the calculation algorithm provided within the tool.

For cohort studies, the ROBINS-I tool will be used, which is a tool for assessing RoB in nonrandomized trials for interventions [10]. The 7 domains included in the assessment include bias from confounding, from selecting patients into the study, from classification of intervention, from deviations from intended intervention, from missing data, from the measurement of outcomes, and from the selection of reported outcomes. The overall risk will also be calculated using the algorithms provided within the tool itself.

### Data Items

Our prepiloted data extraction form will include the following data items:

- Bibliometric data: authors, year of registration, trial registration number, and digital object identifier.
- Methodology: number of participating centers, location of the study, method for blinding, randomization methods, treatment setting (inpatient vs outpatient), and follow-up duration.
- Baseline data: total sample size, mean age, sex with number of female patients included, comorbidities, choice of antibiotic, route of administration, frequency and duration of administration, daily and cumulative dosage, and species of organisms identified if performed.
- Outcome data: duration to infection resolution, duration to symptom improvement, incidence of severe infection, mortality, QOL scale and respective score, length of hospitalization, incidence of adverse events, and incidence of *C difficile* infection.

### Statistical Analysis

We will first provide a comprehensive qualitative synthesis of all of the included studies, summarizing their characteristics and findings in organized text and table format in accordance with the PRISMA reporting guidelines.

If there are sufficient similar studies that will be meaningful for an aggregate analysis, we will perform meta-analyses for a weighted effect of the interventions across different studies. We will conduct our quantitative analysis using the computer program Review Manager (RevMan, version 5.4; Cochrane) [11]. We will use a random-effects model, and in cases where the heterogeneity cannot be explained, we will perform a sensitivity analysis using a random-effects model as well. For continuous outcomes such as duration of infection resolution and length of hospitalization, a mean difference with a 95% CI will be used. For dichotomous outcomes such as mortality or incidence of severe infection, a Mantel-Haenszel odds ratio with 95% CIs will be used. If the events are rare, a Peto odds ratio will be used instead. If there are not enough meaningful data for meta-analysis for any particular outcome, we will qualitatively describe the results across studies.

The heterogeneity of the included studies will be assessed using a combination of visual inspection of the forest plots along with the *I*^2^ statistic according to the Cochrane Handbook. We will consider an *I*^2^>50% to be seriously heterogeneous and an *I*^2^>75% to be very seriously heterogeneous.

In the case of missing data, we will attempt to contact the authors of the original study. Missing SDs will be inputted using methods outlined in the Cochrane Handbook for Systematic Review of Interventions using correlation coefficients.

Regarding the overall assessment of the quality of evidence included in the review, 2 independent reviewers will perform the GRADE (Grading of Recommendations, Assessment, Development, and Evaluation) analysis [12]. Any discrepancies will also be resolved through discussion.

### Ethical Considerations

Ethics approval is not applicable to this study as this is a review study and no original data pertaining to humans or animals will be collected. No individual patient data are collected and all research data collected from original studies will remain anonymized.

## Results

We will share the results of this study through peer-reviewed academic publications and conference presentations. We will continue to perform updating the searches in all databases every 2 months for any new data to be included in the systematic review. This project received no funding or financial assistance. Data analysis is currently underway, and the results are expected to be submitted for publication in late November 2023.

## Discussion

This protocol outlines the planned systematic review and meta-analysis to compare the efficacy of oral and IV antibiotic administration in the treatment of cellulitis. Because of the vast selection of options available to physicians and the lack of quality evidence or rigorous reviews, there has been significant variation between guidelines and physician preferences. Some antibiotics may be available for both IV and oral administration, while others may only be suitable for one of the routes. Therefore, we also hope to aggregate the evidence from the literature first and then elucidate the indications for the usage of either route of administration, potentially also comparing their usage for similar infection profiles to assess their efficacy.

Our review will have several strengths and weaknesses. In terms of strengths, we will not be placing any language or time restrictions in our search strategy to ensure broad inclusion of all currently available evidence. We will also include both prospective and retrospective cohort studies in addition to RCTs to increase our pooled sample size. As a weakness, the quality of our meta-analysis may be limited by our inclusion of cohort studies, which is inherently weaker on the hierarchy of evidence with a potentially higher RoB. However, to mitigate this, we will analyze RCTs and cohort studies in separate pools, as well as using tools such as RoB 2.0 and ROBINS-I to rigorously assess the quality of the included studies based on the Cochrane Handbook. We will also conduct a subgroup analysis with only high-quality evidence and compare those with the pooled results. Additionally, because of the variations that exist within guidelines and physician practices, we also expect potentially significant heterogeneity between our included studies. We will assess this heterogeneity quantitatively and report on it in our final review. An additional limitation of our review is that, despite placing no language restrictions, the databases searched are primarily Western, English-based databases, meaning additional non-English data may be missed. We are only focusing on adults aged 18 years or older; hence, the value of this study for patients aged 18 years or younger may be limited.

There have been several attempts at assessing the different routes of antibiotic administration for the treatment of cellulitis. Brindle et al [3] conducted a systematic review on cellulitis treatments with the route of administration as a subfocus, finding that there are only a few low-quality randomized trials comparing the different routes, showing no difference between IV and oral treatments. Cross et al [6], with their primary focus on the duration of therapy, arrived at a similar conclusion. This study hopes to build upon these previous findings, as we have the routes of administration as our primary comparison [6]. We will also include observational studies and place no language barriers in our inclusion criteria, with the hope that it could provide additional valuable data upon Brindle et al’s [3] and Cross et al’s [6] conclusions on oral and IV treatments being equivalent. We hope the results of this study will inform the future prescribing practice of physicians treating cellulitis, especially in deciding oral versus IV treatments. This would not only benefit the patients but also help control the widening spread of antibiotic resistance in the community.

## Appendix

Multimedia Appendix 1 Detailed sample search strategy of MEDLINE through OVID.

Multimedia Appendix 2 Inclusion and exclusion criteria.

## Abbreviations

GRADE: Grading of Recommendations, Assessment, Development, and Evaluation
IV: intravenous
PRISMA: Preferred Reporting Items for Systematic Review and Meta-Analyses
PROSPERO: International Prospective Register of Systematic Reviews
QOL: quality of life
RCT: randomized controlled trial
RoB: risk of bias
RoB 2.0: revised tool for risk of bias in randomized trials
ROBINS-I: risk of bias in nonrandomized studies for interventions

## Data Availability

Data sharing is not applicable to this article, as no data sets were generated or analyzed during this study.
