# Supplementary material for: Comparing Oral Versus Intravenous Antibiotics Administration for Cellulitis Infection: Protocol for a Systematic Review and Meta-Analysis
Source: JMIR Res Protoc. 2023 Nov 3;12:e48342. doi: 10.2196/48342 (PMC10656654; doi:10.2196/48342)
Supplement: Multimedia Appendix 1 [file resprot_v12i1e48342_app1.docx]

Multimedia Appendix 1

Ovid MEDLINE(R) ALL <1946 to February 20, 2023>

1 Cellulitis/

2 Erysipelas/

3 erysipelas.tw,kf.

4 cellulitis.tw,kf.

5 1 or 2 or 3 or 4

6 Anti-Bacterial Agents/

7 (antimicrob* or antibiotic*).tw,kf.

8 6 or 7

9 5 and 8
