# Supplementary material for: Comparing Oral Versus Intravenous Antibiotics Administration for Cellulitis Infection: Protocol for a Systematic Review and Meta-Analysis
Source: JMIR Res Protoc. 2023 Nov 3;12:e48342. doi: 10.2196/48342 (PMC10656654; doi:10.2196/48342)
Supplement: Multimedia Appendix 2 [file resprot_v12i1e48342_app2.docx]

| **Category** | **Inclusion criteria** | **Exclusion criteria** |
| --- | --- | --- |
| Article type | Randomized control trials, prospective and retrospective observational cohort studies. | Cross-sectional studies, case-control studies, case series, case reports. |
| Patient population | Adults ≥ 18 years old who have been diagnosed with cellulitis or erysipelas of any severity, including both inpatient and outpatient. | Patients ≤ 18 years old. Patients who received placebo treatments. Patients who received topical antibiotic treatments. |
| Intervention | Oral administration of antibiotics. Antibiotics of all classes are included. | Placebo or topic treatments. Antifungals are excluded. |
| Comparator | Intravenous administration of antibiotics. All classes are included. | Placebo or topic treatments. Antifungals are excluded. |
| Outcome | Primary: duration to infection resolution.  Secondary: incidence of severe infection, sepsis, mortality, adverse events and side effects. | No outcomes will be excluded in the screening stage. |

**Table S1**. Inclusion and exclusion criteria.
